# Plant-Derived Alkaloids: The Promising Disease-Modifying Agents for Inflammatory Bowel Disease

**DOI:** 10.3389/fphar.2019.00351

**Published:** 2019-04-12

**Authors:** Jiao Peng, Ting-Ting Zheng, Xi Li, Yue Liang, Li-Jun Wang, Yong-Can Huang, Hai-Tao Xiao

**Affiliations:** ^1^Department of Pharmacy, Peking University Shenzhen Hospital, Shenzhen, China; ^2^School of Pharmaceutical Sciences, Health Science Center, Shenzhen University, Shenzhen, China; ^3^The Key Laboratory of Pharmacology and Druggability for Natural Medicines, Department of Education, Guizhou Medical University, Guiyang, China; ^4^Shenzhen Key Laboratory for Drug Addiction and Medication Safety, Peking University Shenzhen Hospital, Shenzhen Peking University–The Hong Kong University of Science and Technology Medical Center, Shenzhen, China; ^5^Department of Ultrasound Imaging, Peking University Shenzhen Hospital, Shenzhen, China; ^6^Department of Gastroenterology, Peking University Shenzhen Hospital, Shenzhen, China; ^7^Shenzhen Engineering Laboratory of Orthopaedic Regenerative Technologies, Orthopaedic Research Center, Peking University Shenzhen Hospital, Shenzhen, China

**Keywords:** inflammatory bowel disease, inflammation, plant-derived alkaloids, therapeutic effects, action mechanism

## Abstract

Inflammatory bowel disease (IBD) represents a group of intestinal disorders with self-destructive and chronic inflammation in the digestive tract, requiring long-term medications. However, as many side effects and drug resistance are frequently encountered, safer and more effective agents for IBD treatment are urgently needed. Over the past few decades, a variety of natural alkaloids made of plants or medicinal herbs have attracted considerable interest because of the excellent antioxidant and anti-inflammatory properties; additionally, these alkaloids have been reported to reduce the colonic inflammation and damage in a range of colitic models. In this review paper, we summarize the recent findings regarding the anti-colitis activity of plant-derived alkaloids and emphasize their therapeutic potential for the treatment of IBD; obvious improvement of the colonic oxidative and pro-inflammatory status, significant preservation of the epithelial barrier function and positive modulation of the gut microbiota are the underlying mechanisms for the plant-derived alkaloids to treat IBD. Further clinical trials and preclinical studies to unravel the molecular mechanism are essential to promote the clinical translation of plant-derived alkaloids for IBD.

## Introduction

Inflammatory bowel disease (IBD) is commonly divided into two disorders: Ulcerative colitis (UC) and Crohn’s disease (CD), which were first described by Sir Samuel Wilks in 1859 and Doctor Crohn in 1932, respectively (Su et al., 2018). UC and CD are characterized by a chronic clinical course of relapse and remission associated with self-destructive inflammation in the gastrointestinal tract (Nishimura et al., 2009). In clinics, typical symptoms were shown to include weight loss, frequent bowel movement, bloody diarrhea, abdominal pain and urgency (Xiao et al., 2013). The incidence and prevalence of IBD was traditionally high in North America and Europe, and relatively rare in Asia (Sýkora et al., 2018). However, with rapid industrialization in past decades, the present incidence of IBD is also rising rapidly in Asia, paralleling with Westernization. It is reported that the incidence of IBD in the Western world has risen to 0.5% of the general population, and the population of IBD patients in China and India now exceeds one billion (Kaplan, 2015), which places IBD as a global disease.

Despite enormous clinical and experimental studies focusing on the development of IBD in both humans and experimental animals, during the past several decades, the precise etiology of IBD has remained elusive. Most scholars regard it is a complex disease caused by a combination of genetics, immune responses, enteric flora, and environmental factors (Xiao et al., 2015). Current strategies for the treatment of IBD involve inducing and maintaining remission, and current medical therapies in clinics typically involve aminosalicylates, corticosteroids, immunomodulators, and biological agents, which have various side effects that limit their therapeutic benefits (Moreau and Mas, 2015). Aminosalicylates and corticosteroids are commonly used in mild-to-moderate UC patients; however, long-term treatment with these drugs is slightly effective and has side effects such as depression, growth retardation, osteoporosis, and hypertension (Talley et al., 2011). Immunomodulators such as azathioprine and 6-mercaptopurine have serious side effects such as bone marrow suppression, pancreatitis, and hepatic toxicity (Coskun et al., 2016). Infliximab is the most often used biological agent for IBD; however, this agent is expensive and the clinical response is only around 65%. This agent also comes with the risk of infusion-related reactions, lupus-like syndrome and infections-sepsis (Talley et al., 2011; Zhao et al., 2017). The disadvantages of the above-mentioned pharmacological therapies indicate that current therapeutic options are insufficient, thus highlighting that new effective and economic therapies with minimal side effects remains an area of unmet medical need in IBD clinics.

## Plant-Based Alkaloids Against IBD

Alkaloids are a class of amino acid-derived nitrogen-containing organic compounds with low molecular weight, which are mainly contained in various living organisms, such as bacteria, fungi, plants, and animals (Moreira et al., 2018). In plants, alkaloids are secondary metabolites produced in response to environmental modulations and biotic or abiotic stress, which endows alkaloids to have structure diversity and significant biological activities (Taha et al., 2009). These properties make alkaloids potential candidate compounds for new drug development that are thus attracting the increasing attention of scientists. According to their carbon skeletons, alkaloids can usually be classified as indole-, isoquinoline- and pyridine-like alkaloids. However, they can also be classified as ornithine, lysine, tyrosine, and tryptophan-originated alkaloids according to their biochemical precursors (Cushnie et al., 2014). An increasing number of recent studies have reported that alkaloids are effective for treating intestinal inflammatory disorders (Lv Q. et al., 2015; Chen Q. et al., 2017; Chen Y.-Y. et al., 2017; Fu et al., 2017; Zhang X.-J. et al., 2018) and bring good ground for hope for IBD drug development. Therefore, we searched the articles published between 1995 and September 2018 in PubMed/Medline database, using different combinations of keywords including ‘alkaloid,’ ‘inflammatory bowel disease,’ ‘IBD,’ ‘colitis,’ and ‘intestinal inflammation.’ Our thorough and careful search met the following criteria: (i) the focus is on a single compound belonging to alkaloids, (ii) pharmacological studies on *ex vivo* and *in vivo* animal models or human studies, and (iii) only English language articles. The following section summarizes widely studied natural alkaloids that provide treatment against colitis.

### Quinolizidine Alkaloids

Quinolizidine alkaloids are a group of alkaloids possessing a quinolizidine ring or a piperidine ring with diversified pharmacological properties, including cytotoxic, oxytocic, antipyretic, antibacterial, antiviral, and hypoglycemic activities (Bunsupa et al., 2012). Sophocarpine (**1**) and sophoridine (**2**) are two active quinolizidine alkaloids isolated from *Sophora alopecuroides*, which distributes in western and central Asia and been used as traditional Chinese medicine for the treatment of bacterial infections, fever, rheumatism, and cardiovascular diseases (Zhang Y.-B. et al., 2018). Studies of dextran sodium sulfate (DSS)-induced murine colitis have revealed that oral administration of sophocarpine (15, 30, and 60 mg/kg of body weight) significantly ameliorates DSS-induced colitis, which was associated with a reduction of serum IL-1β and IL-6 levels and colonic myeloperoxidase (MPO) activity (Wang et al., 2012), and sophoridine treatment (25 and 50 mg/kg of body weight, p.o.) has shown similar beneficial effects through reducing elevated plasma haptoglobin (HP) and colonic intercellular adhesion molecule-1 (ICAM-1) gene expression, and maintaining the level of cecum immunoglobulin A (sIgA) (Zhao et al., 2010). As a stereoisomer of sophoridine, matrine (**3**), an alkaloid found in kinds of Sophora plants, such as *Sophora moorcroftiana* and *Sophora alopecuroides*, was expected to have similar protective effects on trinitrobenzene sulfonic acid (TNBS)-induced acute colitis and spontaneously developed chronic colitis in mice (Cheng et al., 2006). Cheng et al. (2006) reported that the mice that received matrine (10 and 20 mg/kg of body weight, p.o.) showed significantly improved TNBS-induced colitis by reducing the up-regulated production of colonic TNF-α. Additionally, Wu et al. (2016) reported that in spontaneously developed chronic colitis in mice, the treatment of matrine (10 mg/kg of body weight, p.o.) effectively promoted the recovery of colitis by reducing IL-12/23p40, IFN-γ and IL-17 secretion, lowering the proportion of CD4-positive cells, and inhibiting IFN-γ and IL-17 mRNA expression. Oxymatrine (**4**) is a natural *N*-oxide derivative of matrine, which exists in some kinds of Sophora plants, including *Sophora moorcroftiana, Sophora alopecuroides*, and *Sophora flavescens* (Xia et al., 2011). Like studies of matrine, studies of oxymatrine have revealed that oxymatrine (63 mg/kg of body weight in rats, p.o.) significantly attenuates the colonic injury of TNBS-induced colitis, which is correlated with the regulation of an imbalance of Th1/Th2 cytokines to decrease the level of colonic IL-2 and increase the level of colonic IL-10. In addition, this beneficial effect is associated with the suppression of β2-adrenergic receptors (β2AR), β-arrestin-2 and p65 NF-κB subunit expression in the spleen and colonic tissues (Fan et al., 2008, 2012). In parallel, Guzman et al. (2013) studying the nature of DSS-induced murine colitis revealed that oxymatrine (10 mg/kg of body weight, i.p.) could ameliorate the overall intestinal inflammation by blocking LPS-induced NF-κB nuclear translocation and activity, which is independent of IκBα degradation/phosphorylation. Chen Q. et al. (2017) also found that oxymatrine (25, 50, and 100 mg/kg of body weight in mice, i.p.) exhibited this beneficial effect by inhibiting colonic Th1 and Th17 cell responses via the PI3K/AKT pathway. As well, several studies investigated the toxicities of sophocarpine, matrine, and oxymatrine, and found the median lethal dose (LD50) value of sophocarpine on intravenous injection in mice was 63.94 mg/kg (Qian et al., 2012), the LD50 value of matrine on intraperitoneal administration in mice was 157.13 mg/kg (Wang et al., 2010), as well the LD50 value of oxymatrine in male and female mice by intraperitoneal route were 347.44 and 429.15 mg/kg, respectively (Shi et al., 2018).

**FIGURE 1:**
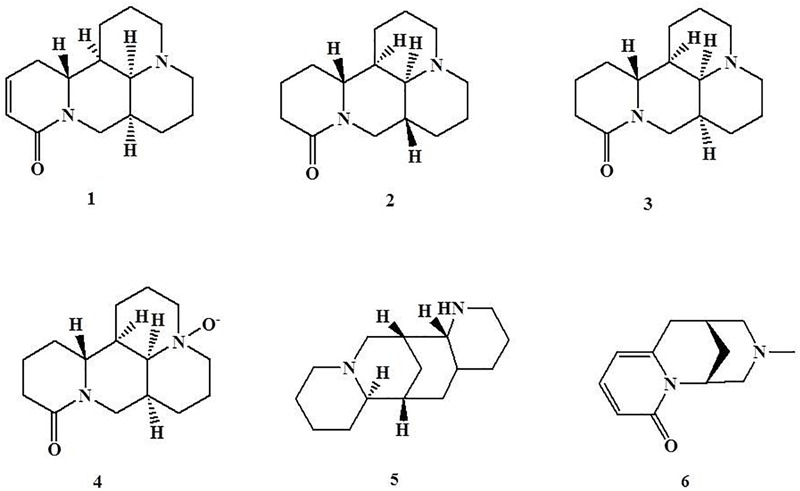
Chemical structures of compounds **1–6**.

Like sophocarpine, sophoridine, matrine and oxymatrine, aloperine (**5**) is a tricyclic quinolizidine alkaloid extracted from traditional Chinese medicine *Sophora alopecuroides*. Fu et al. (2017) studied the protective effects of aloperine on DSS-induced colitis in experiments with mice and reported that oral administration of aloperine (40 mg/kg of body weight) effectively attenuated the severity of DSS-induced colitis by reducing T-cell proportions and increasing Foxp3 expression in the spleen and mesenteric lymph nodes, as well as suppressing p-PI3K p85, p-Akt, and p-mTOR expression and increasing protein phosphatase 2A (PP2A) expression in the colon of colitic mice. An *in vitro* study demonstrated that aloperine (0.5 and 1 mM) can inhibit Jurkat and mouse naïve T-cell apoptosis by inhibiting PI3K/Akt/mTOR signaling, but this beneficial effect can be reversed by PP2A inhibitor, LB-100. These findings indicated that aloperine can regulate inflammatory responses in colitis by inhibiting PI3K/Akt/mTOR signaling in a PP2A-dependent manner (Fu et al., 2017). *N*-methylcytisine (**6**) is also a tricyclic quinolizidine alkaloid isolated from the seeds of traditional Chinese medicines *Laburnum anagyroides* and *Sophora alopecuroides.* It has been reported that oral administration of *N*-methylcytisine (4 and 16 mg/kg of body weight in mice) significantly attenuates the DSS-induced clinical symptoms and pathological damage which are associated with inhibiting colon proinflammatory cytokines through down-regulating NF-κB activation by inhibiting IκB and IKK phosphorylation (Jiao et al., 2018) (Figure 1).

### Isoquinoline Alkaloids

Isoquinoline alkaloids are classes of medicinally active alkaloids formed from a precursor of 3,4-dihydroxytyramine (dopamine) linked to an aldehyde or ketone with multiple properties including antispasmodic, antimicrobial, antitumor, antifungal, anti-inflammatory, cholagogue, hepatoprotective, antiviral, amebicidal, and anti-oxidant activities (Iranshahy et al., 2014). Berberine (**7**), a well-known representative of this type of alkaloid mainly isolated from traditional Chinese medicines *Coptis chinensis* and *Phellodendron chinense*, has been reported to possess the ability to protect experimental colitis through regulating innate and adaptive immune responses, intestinal barrier function and the gut microbiota (Lee et al., 2010; Yan et al., 2012; Kawano et al., 2015; Li et al., 2015, 2016; Lv Z. et al., 2015; Zhang et al., 2017; Cui et al., 2018; Jing et al., 2018; Liu et al., 2018). Among these reports, Yan et al. (2012) revealed that oral administration of berberine (100 mg/kg of body weight in mice) significantly ameliorated DSS-induced intestinal injury and colitis associated with decreasing the disruption of barrier function and apoptosis of colon epithelium, thus inhibiting the proinflammatory cytokine TNF-α, IFN-γ, KC, and IL-17 production of colonic macrophages and promoting the apoptosis of colonic macrophages through down-regulating the activation of MAPK and NF-κB (Yan et al., 2012). Zhang et al. (2017) also observed the effect of berberine on the intestinal barrier function of DSS-induced murine colitis and found that berberine (100 mg/kg of body weight, p.o.) significantly inhibited the increase of fluorescein isothiocyanate-dextran in serum and the decrease of zonula occluden-1, occluding, and epithelial cadherin expression in colonic tissue. Liu et al. (2018) investigated the effect of berberine on macrophage polarization in experimental colitis and observed that berberine (40 mg/kg of body weight in mice, p.o.) could significantly reduce the percentage of M1 macrophages, and this beneficial effect was associated with activating the AKT1/SOCS1 signaling pathway to inhibit p65 NF-κB subunit phosphorylation. Li et al. (2015) also investigated the effect of berberine on the immune responses of Th1 and Th17 cells in experimental colitis and showed that berberine (50 and 100 mg/kg of body weight in mice, p.o.) could adjust the ratio of M2/M1 macrophages and suppress the number of Th1 and Th17 cells through down-regulating the phosphorylation of STAT1, STAT3, and NF-κB, but had no effect on regulatory T cells (Tregs); this similar action mechanism was observed in DSS-induced chronic relapsing colitis (Li et al., 2016). Notably, Kawano et al. (2015) revealed that berberine was the antagonist of dopamine D1- and D2-like receptors, which (40 μg/mouse, i.p.) could significantly inhibit the dendritic cell-mediated generation of Th1 and Th17 subsets that depend on dopamine receptor antagonist-mediated suppression. In addition, Lee et al. (2010) revealed that berberine (10 and 20 mg/kg of body weight in mice, p.o.) could significantly reduce the number of *Enterobacteriaceae* and restore the number of *Bifidobacteria* reduced by TNBS. Lv Z. et al. (2015) reported that berberine administration (100 mg/kg of body weight in mice, p.o.) could promote the restoration of the intestinal microbiota by inhibiting the expansion of members of the family *Enterobacteriaceae* and counteracting the side effects of vancomycin in treatment of the relapse of *Clostridium difficile* infection. In addition, Cui et al. (2018) found berberine (40 mg/kg of body weight in mice, p.o.) could reduce the diversity of the gut microbiota and interfered with the relative abundance of *Desulfovibrio, Eubacterium*, and *Bacteroides.* Fecal transplantation from berberine-treated mice could relieve UC and regulate the Treg/Th17 balance. Further, using DSS-induced murine colitis, Jing et al. (2018) investigated the effect of berberine on P-glycoprotein (P-gp), one of the most important proteins of the cell membrane that pumps harmful molecules out of the intestinal mucosa; the results revealed that berberine (10 and 40 mg/kg of body weight in mice, p.o.) could significantly ameliorate intestinal inflammation by enhancing P-gp expression, which is independent of nuclear factor erythroid 2-related factor 2 (Nrf2) activation. Several workers have reported berberine was low toxicity (Rad et al., 2017; Singh and Sharma, 2018). The LD50 value of pure berberine on intraperitoneal and orally administration in mice were 23 and 329 mg/kg, respectively. Moreover, the LD50 value of berberine by intraperitoneal administration was equal to 205 mg/kg (Singh and Sharma, 2018).

Berberrubine (**8**), a naturally occurring isoquinoline alkaloid, is also one of the main metabolites of berberine *in vivo* (Spinozzi et al., 2014). Both berberine and berberrubine can be found in the medicinal plant *Berberis vulgaris*, a Iranian traditional medicine to cure jaundice, enlarged liver, enlarged spleen, eye sores, toothache, asthma, and skin pigmentation, etc. (Rahimi-Madiseh et al., 2017; Yu et al., 2018). Compared to berberine, berberrubine has more hydrophily and higher plasma concentration after berberine oral administration owing to its more efficient intestinal absorption (Zuo et al., 2006). Yu et al. (2018) studied the anti-colitis effect of berberrubine on murine colitis model. The results showed that mice that received berberrubine (10 and 20 mg/kg of body weight in mice, p.o.) could significantly improve DSS-induced colitis by reducing the disease activity index, alleviating inflammatory cell infiltration, inhibiting MPO activity and cytokines (TNF-α, IFN-γ, IL-1β, IL-6, and IL-4) production, upregulating the expression of tight junction (TJ) proteins (zonula occludens-1, zonula occludens-2, claudin-1, and occludin) and mRNA expression of mucins, and decreasing the Bax/Bcl-2 ratio; the beneficial effect is comparable to berberine (50 mg/kg of body weight in mice, p.o.), indicating that berberrubine possesses a pronounced anti-colitis effect similar to berberine (Yu et al., 2018).

Similar to the structures of berberine and berberrubine, palmatine (**9**), demethyleneberberine (**10**), sanguinarine (**11**) and cavidine (**12**) structures are also naturally occurring isoquinoline alkaloids. Palmatine (**9**) can be found in many plants such as *Phellodendron amurense, Coptis chinensis*, and *Corydalis yanhusuo*. Zhang X.-J. et al. (2018) reported that in DSS-induced colitis during the experiments with mice, the treatment of palmatine (50 and 100 mg/kg of body weight in mice, p.o.) effectively promoted the recovery of colitis by improving mucosal integrity, inhibiting epithelial cell apoptosis, increasing the relative abundance of *Bacteroidetes* and *Firmicutes*, and reducing the amount of *Proteobacteria*, as well as suppressing tryptophan catabolism. Demethyleneberberine (**10**) is an active component of *Phellodendri chinensis*, a traditional Chinese medicine widely used for the treatment of diabetes, tumors, and inflammatory diseases (Cao et al., 2018). Chen Y.-Y. et al. (2017) reported that the mice that received demethyleneberberine (150 and 300 mg/kg of body weight in mice, p.o.) could significantly attenuate the colonic injury of DSS-induced colitis by inhibiting the NF-κB pathway and regulating the balance of Th cells. Sanguinarine (**11**) and cavidine (**12**) are mainly obtained from the roots of *Sanguinaria canadensis*, a traditional medicine used by Native Americans, and *Corydalis impatiens*, a folk medicine used in Tibet, respectively (Niu X. et al., 2015; Croaker et al., 2016). Studies in acetic acid-induced colitis revealed that sanguinarine (1, 5, and 10 mg/kg of body weight in mice, p.o.) or cavidine (1, 5, and 10 mg/kg of body weight in mice, p.o.) treatment significantly attenuated colonic mucosal injury by reducing serum and colonic pro-inflammatory cytokines TNF-α and IL-6, and improving colonic oxidative status through down-regulating p65 NF-κB subunit expression (Niu et al., 2013; Niu X.F. et al., 2015). The toxicologic studies indicated that LD50 value of sanguinarine was 1,658 mg/kg in rats by orally administration, 29 mg/kg in rats by intra-venous administration and greater than 200 mg/kg in rabbits by dermis delivery (Singh and Sharma, 2018).

Boldine (**13**) and norisoboldine (**14**) are two natural benzylisoquinoline alkaloids obtained from the roots of *Lindera aggregata*, a traditional Chinese medicine used to treat rheumatoid arthritis (Luo et al., 2010). Pandurangan et al. (2016) reported that the mice orally receiving boldine (50 mg/kg of body weight in mice, p.o.) significantly suppressed the disease symptoms of DSS-induced colitis and reduced colonic TNF-α, IL-6, and IL-17 production and protein expression of p-STAT3 and p65 NF-κB subunit. It was suggested that the beneficial effect of boldine was likely mediated by the inhibition of NF-κB and STAT3 signaling pathways (Pandurangan et al., 2016). Similarly, norisoboldine (20 and 40 mg/kg of body weight in mice, p.o.) showed potent protective effects against DSS-induce colitis in mice, which could markedly reduce the symptoms of colitis, the levels of IL-1β and TNF-α, and the activation of ERK, p38 MAPK, and p65 NF-κB subunit in colonic tissues. Norisoboldine was also found to increase the number of CD4^+^ CD25^+^ Foxp3^+^ Treg cells in mesenteric lymph nodes and colonic lamina proprias and improve Foxp3 mRNA expression and Smad2/3 phosphorylations in colon tissues. *In vitro* studies have also demonstrated that norisoboldine (1, 3, 10, and 30 μM) can facilitate the differentiation of Treg cells (Lv Q. et al., 2015). A molecular mechanism study further revealed that norisoboldine is a natural aryl hydrocarbon receptor (AhR) agonist, which promoted Treg differentiation and alleviated the development of colitis by regulating the AhR/glycolysis axis and the subsequent NAD^+^/SIRT1/SUV39H1/H3K9me3 signaling pathway (Lv et al., 2018a). Additionally, studies of TNBS-induced colitis revealed that treatment with norisoboldine (20 and 40 mg/kg of body weight in mice, p.o.) significantly attenuated colonic mucosal injury by inhibiting NLRP3 inflammasome activation by regulating the AhR/Nrf2/ROS signaling pathway (Lv et al., 2018b). These findings indicate that the molecular mechanisms behind the anti-colitis effect of norisoboldine inhibit NLRP3 inflammasome activation and promote Treg differentiation. Notably, the toxicologic study demonstrated that consecutive oral administration of boldine at 800 mg/kg for 90 days did not cause any obvious histological modification in rats, but only presented minor miscarriage and teratogenic action (Almeida et al., 2000), suggesting boldine is contraindicated in pregnant women.

Tetrandrine (**15**) and neferine (**16**) are two bis-benzylisoquinoline alkaloids obtained from traditional Chinese medicines *Stephenia tetrandra* and *Nelumbo nucifera*, respectively (Zhang et al., 2009). In experiments with the colon of DSS-treated mice, it has been reported that oral administration of tetrandrine (40 mg/kg of body weight in mice) significantly improved the disease activity index and histological score and reduced the NF-κB DNA binding activity, colonic MPO activity, and colonic IL-1β and TNF-α (Zhang et al., 2009). Additionally, Wu et al. (2018) reported that the mice orally receiving neferine (10 and 25 mg/kg of body weight in mice) significantly ameliorated DSS-induced intestinal colitis associated with inhibiting the protein expression of iNOS, COX-2, receptor-interacting protein 1 (RIP1), RIP3, and mixed lineage kinase domain like protein (MLKL), and increasing the protein expression of caspase-8 in colon tissues. The low toxicity of tetrandrine had been clearly stated and the data showed the LD50 value was about 444.67 ± 35.76 mg/kg on intravenous administration in mice (Shi et al., 2016).

**FIGURE 2:**
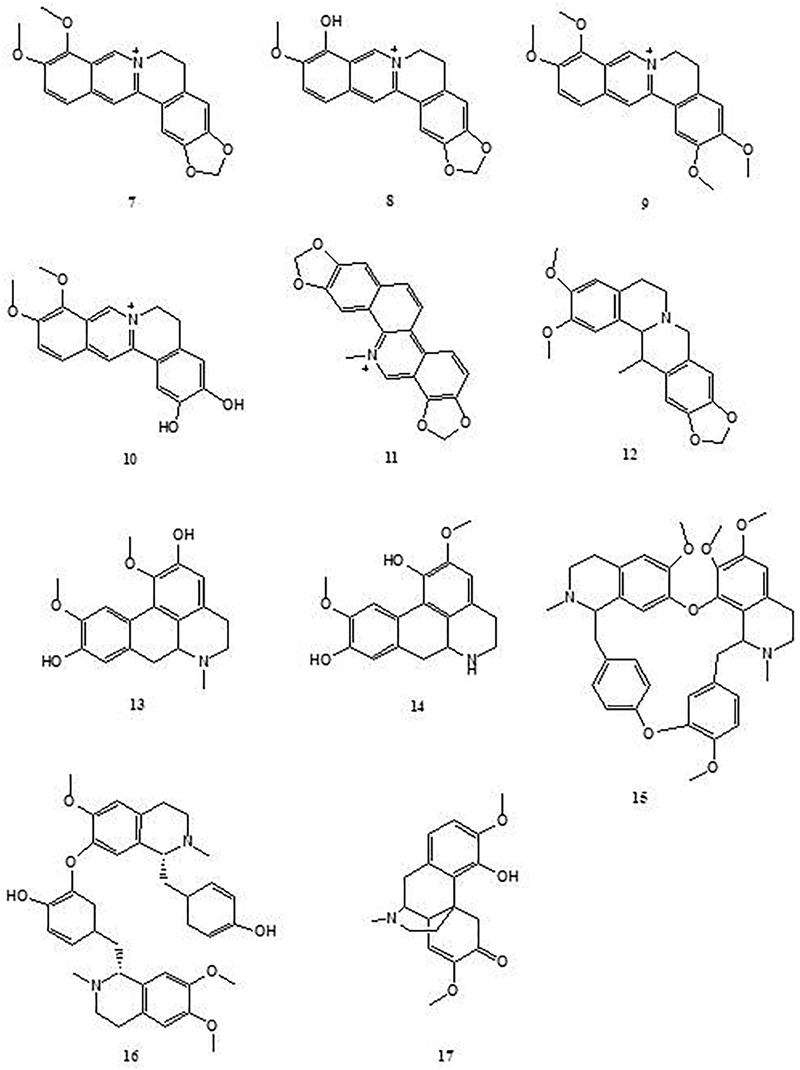
Chemical structures of compounds **7–17**.

Sinomenine (**17**) is a morphinan-type isoquinoline alkaloid, which was isolated from the medical plant *Sinomenium acutum*, a traditional herbal medicine used in Japan, China, and Korea against rheumatism, fever, pulmonary diseases and mood disorders (Yoo et al., 2017). Studies in TNBS-induced colitis revealed that treatment with sinomenine (100 and 200 mg/kg of body weight in mice, p.o.) significantly attenuated the clinical and histopathologic severity of colitis related to the reduction of colonic protein expression of c-Maf, TNF-α, IFN-γ and miR-155, as well as the production of TNF-α and IFN-γ (Cheng et al., 2007; Yu et al., 2013). This beneficial effect was also observed on DSS-induced murine colitis, and a molecular mechanism study further revealed that sinomenine (100 mg/kg of body weight in mice, p.o.) could markedly increase the expression of Nrf2 and its downstream genes, heme oxygenase-1 and NADP(H) quinone oxidoreductase 1 (NQO-1) (Zhou et al., 2018). It was indicated that sinomenine could attenuate colonic mucosal injury by improving the colonic oxidative status via the Nrf2/NQO-1 signaling pathway (Figure 2). In a toxicologic study, Fu et al. (1963) reported that sinomenine has a LD50 value of 580 ± 51 mg/kg on orally administration in mice, and rats receiving sinomenine 694 mg/kg orally showed no significant toxic reactions but presented sedation.

### Indole Alkaloids

Indole alkaloids are classes of alkaloids containing a structural moiety of indole with various biological properties including analgesic, regulation of central and peripheral nervous systems, antimicrobial, anti-ulcer, antioxidant, and antimalarial activities (Singh et al., 2011). Isatin (**18**) is a natural indole alkaloid obtained from *Isatis tinctoria*, an anti-inflammatory and dye medicinal plant in Europe and China (Zhou and Qu, 2011). Rabelo Socca et al. (2014) reported that oral treatment of isatin (6 and 25 mg/kg of body weight in rats) was capable of protecting against TNBS-induced gut mucosa injury, which were associated with decreasing colonic TNF-α, COX-2, and PGE^2^ levels, and MPO activity, and increasing colonic IL-10 and glutathione (GSH) levels, superoxide dismutase (SOD), glutathione peroxidase (GSH-Px), and glutathione reductase (GSH-Rd) activities. Another alkaloid, tryptanthrin, (**19**) is a natural type of indole alkaloid obtained from the medicinal plant *Polygonum tinctorium*, a useful medicinal plant cured for inflammation, allergy, disorders by bacteria and cancer (Lee et al., 2018). Studies in DSS-induced colitis revealed that tryptanthrin (100 mg/kg of body weight in mice, p.o.) treatment significantly attenuated colonic mucosal injury, which is associated with reducing pro-inflammatory mediators PGE2, TNF-α, and nitric oxide (NO) production by regulating the TNF-α/NF-κBp65 and IL-6/STAT3 signaling pathways by inhibiting the degradation of IκBα and the phosphorylation of STAT3 in colon tissues (Micallef et al., 2002; Wang et al., 2018).

**FIGURE 3:**
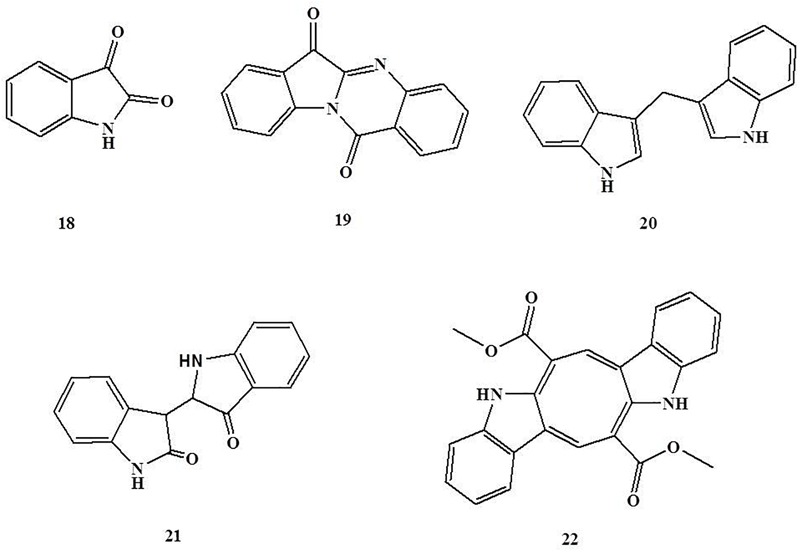
Chemical structures of compounds **18–22**.

In addition, 3,3-diindolylmethane (**20**), a bisindole alkaloid, is mainly produced by acid-catalyzed oligomerization of indole-3-carbinol (I3C) obtained from Brassica food plants. Administration of 3,3-diindolylmethane (10 and 50 mg/kg of body weight in mice, p.o.) has been found to significantly attenuate the severity of TNBS-induced murine colitis, which was associated with the increase of expression of BRCA1, the decrease of reactive oxygen species (ROS) generation, and the inhibition of vascular cell adhesion molecule-1 (VCAM-1) expression and leukocyte-endothelial cell adhesion. It is suggested that the beneficial effect of 3,3-diindolylmethane was medicated by a BRCA1-dependent antioxidant pathway (Huang et al., 2011). In parallel, a study in oxazolone-induced colitis revealed that administration of 3,3-diindolylmethane (50 mg/kg of body weight in mice, i.p.) could effectively attenuate intestinal injury by inhibiting Th2/Th17 cells and promoting Tregs by activating AhR (Huang et al., 2013). Additionally, Jeon et al. (2016) revealed that 3,3-diindolylmethane (20 mg/kg of body weight in mice, p.o.) treatment could significantly suppress the expression of vascular endothelial growth factor (VEGF)-A and VEGF receptor (VEGFR)-2 as well as the expression of VEGF-C, VEGF-D, VEGFR-3, and angiopoietin-2 to mitigate DSS-induced intestinal inflammation and associated angiogenesis and lymphangiogenesis. These findings indicate that 3,3-diindolylmethane could attenuate colonic mucosal injury by inhibiting ROS-induced VCAM-1 expression and leukocyte recruitment, suppressing Th2/Th17 cells and promoting Tregs, as well as inhibiting angiogenesis and lymphangiogenesis. Similarly, Gao et al. (2016) reported that treatment of indirubin (**21**) (10 mg/kg of body weight in mice, p.o.), a bisindole alkaloid obtained from *Isatis indigotica*, a traditional Chinese medicine used for anti-influenza in clinics (Luo et al., 2019), significantly attenuates DSS-induced intestinal injury. This beneficial effect was associated with suppressing CD4^+^T cell infiltration and promoting the generation of Foxp3-expressing Tregs in colon tissues by inhibiting the activation of NF-κB. Caulerpin (**22**), a bisindole alkaloid obtained from the algaes of *Caulerpa sertularioides* and *Caulerpa mexicana*, acts in the same way. Lucena et al. (2018) investigated the protective effect of caulerpin on DSS-induced colitis and found that oral treatment of caulerpin (4 and 40 mg/kg of body weight in mice) significantly abates the severity of DSS-induced murine colitis, which was partly attributed to decreasing the production of TNF-α, IFN-γ, IL-6, IL-17, increasing the production of IL-10, and suppressing NF-κB p65 subunit activation in colon tissues (Figure 3). Indirubin is low toxicity. Subacute toxicity tests showed that the rats orally administration of indirubin 100–400 mg/kg for 30 consecutive days, no significant untoward effects were observed. Moreover, the dogs were given indirubin, 20–40 mg/kg orally for three consecutive months, no significant abnormality was also observed (Ji et al., 1981).

### Xanthine Alkaloids

**FIGURE 4:**
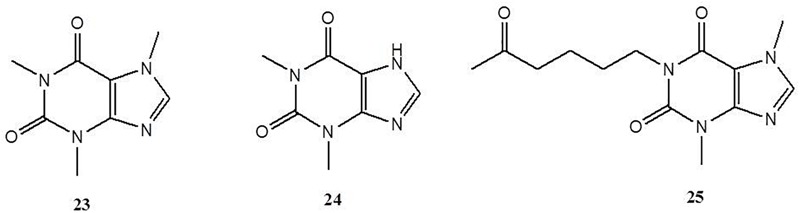
Chemical structures of compounds **23–25**.

Xanthine alkaloids are a group of alkaloids containing xanthine as their nitrogenous base. There is a high concentration of xanthine in coffee, tea, soft drinks, chocolate, kola nuts and certain medicines with various biological properties, such as stimulation of the central nervous system, relaxation of the bronchial smooth muscle, increasing of the heart muscle contractility and efficiency, and suppression of inflammation (Ashihara et al., 2017). Caffeine (**23**) is a representative natural xanthine alkaloid. Lee et al. (2014) revealed that caffeine treatment prevented the onset of colitis with reduced chitinase 3-like 1 expression in intestinal epithelial cells (IECs). *In vitro*, they also found that caffeine (2.5 and 5 mM) treatment in IEC lines significantly down-regulated chitinase 3-like 1 mRNA expression, which resulted in reducing the bacterial invasion in a dose-dependent manner (Lee et al., 2014). Moreover, caffeine-treated colitic mice showed a lower production of pro-inflammatory cytokines and much less bacterial translocation into other organs than the mice in the control group (Lee et al., 2014). Such results indicated that caffeine (5 mM in free drinking water, p.o.) could ameliorate intestinal inflammation through the inhibition of bacterial invasion by reducing chitinase 3-like 1 expression in IECs. Theophylline (**24**) is another natural alkaloid present in tea, which bears structural and pharmacological similarity to caffeine. Ghasemi-Pirbaluti et al. (2017) reported that treatment of theophylline (20 and 50 mg/kg of body weight in mice, i.p.) significantly abates the severity of acetic acid-colitis by reducing colonic MPO activity and TNF-α, IL-1β, and IL-6 concentrations in an inflamed colon. Pentoxifylline (**25**), a xanthine alkaloid found in *Theobroma cacao*, acts in the same way. Experiments with rats suffering from ischemic colitis carried out by Reyhan et al. (2014) showed that orally administered pentoxifylline (50 mg/kg of body weight) significantly downsizes the ischemic area and decreases colonic malondialdehyde (MDA) levels of the rats involved. Likewise, Karatay et al. (2017) investigated the anti-colitis effect of pentoxifylline (100 mg/kg of body weight) on TNBS-induced colitis in rats and found that both intraperitoneal and intrarectal treatment of pentoxifylline could significantly reduce colonic injury by attenuating the accumulation of MDA and TGF-β1 and the activation of MPO, MMP-3, and MMP-1, thus restoring the activity of SOD. Such data indicates that the benefits of pentoxifylline on colitis might be partly mediated by inhibiting oxidative stress and metalloproteinase activities (Figure 4). Notably, the toxicities of caffeine and theophylline should be payed more attention. Lethal doses of caffeine have been reported at blood concentrations of 80 to 100 μg/mL which can be reached with ingestion of approximately 10 g or greater (Murray and Traylor, 2018). As well, theophylline has a narrow therapeutic window range from 10 to 20 μg/mL, even levels slightly above this therapeutic window can lead to adverse effects (Journey and Bentley, 2018).

### Other Alkaloids

Tetramethylpyrazine (**26**) is a natural alkaloid obtained from *Ligusticum chuanxiong*, a well-known traditional Chinese medicine used for the therapy of stroke, angina pectoris, hypertension, and cardiac arrhythmias (Yang et al., 2018). In his oxazolone-induced murine colitis paper, He et al. (2012) reported that treatment with tetramethylpyrazine (100 mg/kg of body weight in mice, i.p.) significantly improves the colonic inflammatory status, decreases the expression of colonic TNF-α, iNOS, NF-κB p65 and COX-2, and increases PPAR-γ production. As inhibition of PPAR-γ signaling would lower the beneficial effect of tetramethylpyrazine, it is suggested that the effect of tetramethylpyrazine treatment in colitis is independent of PPAR-γ. *In vitro* assays also demonstrated that tetramethylpyrazine (40 μg/mL) could significantly inhibit NF-κB translocation, which reduces the production of inflammatory factors TNF-α, IL-6, IL-8, and ROS in LPS treated Caco-2 cells (Lu et al., 2014). These results indicated that tetramethylpyrazine could ameliorate intestinal inflammation by activating the PPAR-γ signaling pathway and inhibiting the NF-κB p65 signaling pathway. 4-methoxy-5-hydroxycanthin-6-one (**27**) is a natural beta-carboline alkaloid obtained from *Picrasma quassioides*, a Chinese traditional folklore medicine used as vermicide, anti-inflammatory and antibacterial agents (Xu et al., 2016). Liu et al. (2009) investigated the anti-colitis effect of 4-methoxy-5-hydroxycanthin-6-one on DSS-induced colitis in experiments with rats and found that 4-methoxy-5-hydroxycanthin-6-one (20 and 40 mg/kg of body weight in rats, p.o.) could significantly mitigate the severity of colitis by preventing colon length shortening and reducing colonic MPO activity and serum TNF-α levels.

14-*O*-acetylneoline (**28**) is a diterpenoid alkaloid isolated from *Aconitum laciniatum*, a Bhutanese traditional medicine responded well to treat chronic infections and inflammatory conditions (Wangchuk et al., 2007; Wangchuk et al., 2015). Recently, Wangchuk et al. (2015) reported that 14-*O*-acetylneoline showed significant protection against TNBS-induced colonic inflammation. Compared to the mice from the colitic control group, mice that received 14-*O*-acetylneoline (10, 20, and 50 μg/mouse, p.o.) showed less weight loss, macroscopic pathology and colonic inflammation and lower colonic mRNA expression of IFN-γ (Wangchuk et al., 2015). Epiisopiloturine (**29**) is an imidazole alkaloid obtained from the leaves of *Pilocarpus microphyllus*, well known in Brazil as jaborandi and used mainly for the treatment of glaucoma (Sawaya et al., 2008). Rodrigues de Carvalho et al. (2018) studied TNBS-induced colitic rats receiving epiisopiloturine, and revealed that epiisopiloturine (1 mg/kg of body weight, p.o.) could significantly suppress the disease symptoms of TNBS-induced acute colitis by reducing the production of pro-inflammatory mediators, IL-1β, NO, and MDA, and expressing inflammatory markers COX-2 and iNOS. Piperine (**30**) is a piperidine alkaloid isolated from the fruits of *Piper nigrum*, a perennial woody climber plant that yields the commercial product known as “black pepper,” as well as a folk medicine in India, Bangladesh, Pakistan, etc. (Thiengsusuk et al., 2018; Takooree et al., 2019). It has been reported that piperine (5 and 10 mg/kg of body weight in mice, p.o.) treatment could effectively prevent colonic inflammation of acetic acid-induced colitis, which was associated with inhibiting pro-inflammatory mediators NO, TNF-α and free fatty acids (FFAs) production (Gupta et al., 2015). Hu et al. (2015) further revealed that the anti-colitis effect of piperine is independent of PXR activation, evidencing by the anti-colitis effect of piperine can be reversed by tail vein injection of PXR small interfering RNA (siRNA). The relative safe characteristic of piperine have been clearly stated in the toxicologic experiments (Piyachaturawat et al., 1983; Chinta et al., 2017; Bastaki et al., 2018). In adult male mice the LD50 value of piperine on intravenous, intraperitoneal, intramuscular, intragastric and subcutaneous orally administration were 15.1, 43, 400, 330, and 200 mg/kg, respectively; In parallel, the LD50 value of piperine on intraperitoneal route in adult female mice and weanling male mice were 60 and 132 mg/kg, respectively. As well, in adult femate rats, the LD50 value of piperine on intraperitoneal administration was 33.5 mg/kg whereas was increased to 514 mg/kg on intragastric route (Piyachaturawat et al., 1983). Bastaki et al. (2018) performed 90-day Good Laboratory Practice (GLP) compliant dietary study of piperine in rats and found that no adverse effects were observated when the rats giving piperine up to 50 mg/kg.

**FIGURE 5:**
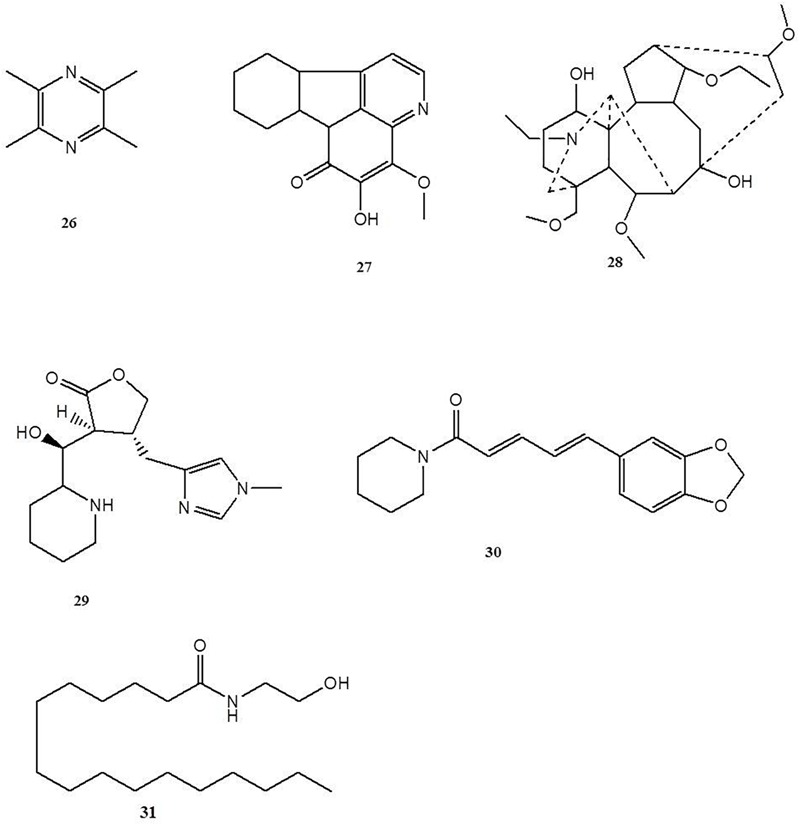
Chemical structures of compounds **26–31**.

Palmitoylethanolamide (31) is an amide alkaloid presented in foods such as egg yolks, peanuts, and soy seeds (Borrelli et al., 2015; Ohara et al., 2018). Borrelli et al. (2015) reported that the mice receiving palmitoylethanolamide (1 mg/kg of body weight in mice, i.p.) treatment could significantly attenuate dinitrobenzene sulfonic acid (DNBS)-induced colonic inflammation and intestinal permeability, stimulate colonic cell proliferation, which is independent of the activation of TRPV1 and CB2 receptor. Capasso et al. (2014) examined the effect of palmitoylethanolamide on intestinal transit in oil of mustard (OM)-induced intestinal inflammation during experiments with mice. It was found that palmitoylethanolamide (1 and 10 mg/kg of body weight in mice, i.p.) treatment could significantly inhibit OM-induced increase in transit. While this effect can be blocked by CB1 receptor antagonist rimonabant, and be elevated by the TRPV1 channel antagonist I-RTX, suggesting CB1 receptors and TRPV1 channels mediated the effect of palmitoylethanolamide on intestinal motility (Capasso et al., 2014). Esposito et al. (2014) also studied the effect of palmitoylethanolamide on enteric glia activation in DSS-induced colitis in experiments with mice and UC patients. The results showed that palmitoylethanolamide (2 and 10 mg/kg of body weight in mice, p.o.) treatment could effectively attenuate intestinal inflammation of colitic mice and UC patients, which was attributed to blocking the S100B/TLR4 axis of enteric glial cells and subsequently inhibiting NF-κB activation (Esposito et al., 2014). However, this effect can be abolished by antagonists of PPARα, but not PPARγ. Notably, Sarnelli et al. (2016) also found that palmitoylethanolamide (2 and 10 mg/kg of body weight in mice, p.o.) could effectively inhibit colitis-associated angiogenesis and decrease VEGF release and new vessels formation, which can be abolished by antagonists of PPARα, but not PPARγ at the same time. These results indicate that palmitoylethanolamide could inhibit enteric glia activation and angiogenesis in a selective PPAR-α dependent mechanism (Figure 5).

### Therapeutic Mechanisms of PLANT-BASED ALKALOIDS for IBD

**FIGURE 6:**
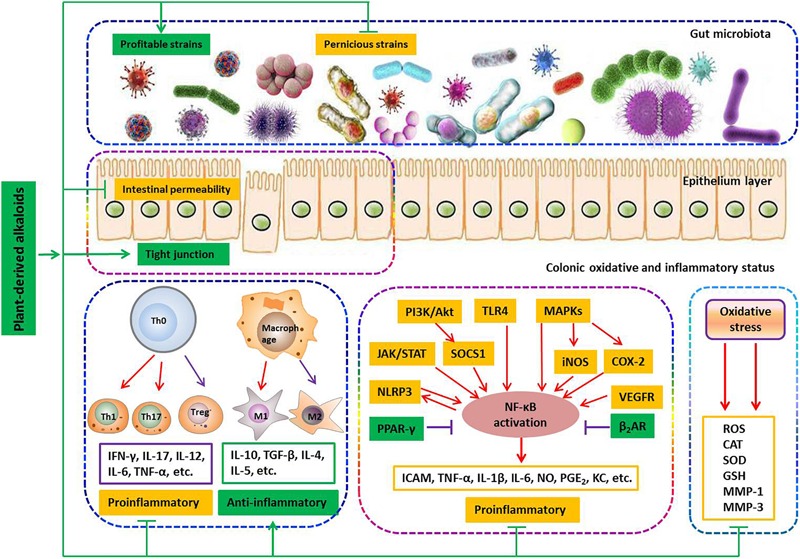
Involved mechanisms of plant-derived alkaloids in treatment of IBD (Promotion ↑, inhibition ⊥).

Current studies revealed that gut microbiota dysbiosis, epithelial barrier impairment, overproduction of ROS, and the imbalance in pro-inflammatory and anti-inflammatory cytokines are associated with the development of colitis. Based on this knowledge, various strategies were proposed. During experiments with animals, a number of plant-based alkaloids seemed to be promising in limiting intestinal inflammation (Figure 6).

### Modulation of Gut Microbiota

It is well-known that the gut microbiota in healthy individuals provides a number of health benefits to the host, relating to pathogen protection, nutrition, metabolism and the immune system (Nishida et al., 2018). The symbiotic interaction between the human host and the microbiota is necessary to maintain human health (Bernstein and Forbes, 2017). An unfavorable alteration of the composition and function of the gut microbiota is known as dysbiosis, which has repeatedly been observed in IBD patients and is now recognized as a key pathologic factor in the development of colitis (Nishida et al., 2018). Dysbiosis of the gut microbiota is characterized by a significant reduction of obligate anaerobes such as members of the phyla *Bacteroidetes* and *Firmicutes* and a sharp increase in facultative anaerobes such as the phyla *Actinobacteria* and *Proteobacteria*, like *Escherichia coli* (Sunkara et al., 2018). A decrease in obligate anaerobes or an increase of facultative anaerobes is associated with reducing the production of inhibitory substances, such as short-chain fatty acids, causing increased inflammation (Rigottier-Gois, 2013). Therefore, dysbiosis correction has been considered an attractive therapeutic approach. It has been reported that both isoquinoline alkaloids berberine (**7**) and palmatine (**9**) could effectively promote the recovery of colitis by increasing the relative abundance of *Bacteroidetes* and *Firmicutes* and reducing the amount of *Desulfovibrio* and *Proteobacteria*. Berberine (**7**) can also confront *Clostridium difficile*-induced colitis by inhibiting the family *Enterobacteriaceae*.

### Restoration of Epithelial Barrier Function

The intestinal epithelial barrier controls the passage of nutrients, water and electrolytes while impeding the passage of pathogens and toxins into sub-epithelial tissues and the internal milieu (Odenwald and Turner, 2017). This barrier is composed of a layer of mucins produced by specialized epithelial cells termed goblet cells and the epithelium itself, which is formed by epithelial cells held together by the apical junctional complex, consisting of adherent junctions and TJs (Marchiando et al., 2010). TJs are composed of transmembrane proteins (claudins and junctional adhesion molecules) and cytosolic scaffold proteins such as zonula occludens (ZOs) (Odenwald and Turner, 2017). A compromised intestinal barrier characterized as increased intestinal permeability, obvious crypt damage and/or decreased expressions of transmembrane proteins is commonly observed in the IBD (Ma et al., 2017). It has been reported that isoquinoline alkaloid berberine (**7**) could significantly suppress DSS-induced increase of intestinal permeability and elevate the expression of zonula occluden-1, occluding and epithelial cadherin in colonic tissue. In parallel, it was found that berberrubine (**8**) significantly upregulates the expression of TJ proteins such as zonula occludens-1, zonula occludens-2, claudin-1 and occludin as well as the mRNA expression of mucins in the colon tissues of DSS-induced colitis mice. It has also been reported that palmatine (**9**) effectively promotes the recovery of colitis by improving mucosal integrity and inhibiting epithelial cell apoptosis. Notably, it was found that xanthine alkaloid caffeine **(23**) significantly down-regulates the chitinase 3-like 1 mRNA expression of IECs, which could greatly inhibit the bacterial invasion into sub-epithelial tissues and the internal milieu. These findings indicate that plant-based alkaloids have potential anti-colitis effects by regulating an epithelial barrier function.

### Regulation of Colonic Oxidative and Inflammatory Status

Oxygen is essential for the normal life of aerobic organisms. Owing to its high redox potential, oxygen would inevitably be translated into ROS such as superoxide anion, hydroxyl radical and hydrogen peroxide, which are known as signal mediators involving in growth, differentiation, progression and death of the cell. However, excessive ROS can interact with biological molecules and generate by-products such as peroxides and aldehydes, which can cause damage to the architecture and function of cells. Under physiological conditions, the generation of ROS is controlled by the antioxidant system, which consists of enzymes including enzymatic antioxidants, such as SOD, catalase (CAT) and GSH-Px, and non-enzymatic antioxidants, such as GSH and vitamins. However, when detrimental stress compromises the antioxidant defense system, a fraction of ROS may escape the intrinsic clearance machinery and induce a state of oxidative stress, leading to cell dysfunction. Substantial evidence suggests that chronic intestinal inflammation is associated with enhanced production of ROS, since ROS could regulate distinct signaling pathways (NF-κB, MAPKs, PI3K/Akt, Nrf2/HO-1, STAT-3/HIF-1α, etc.) to induce the production of pro-inflammatory mediators such as TNF-α, IL-1β and IL-6, which subsequently activate the responses of innate and adaptive immune cells to generate more pro-inflammatory cytokines and ROS. This cascade reaction results in a self-sustaining and autoamplifying vicious circle to worsen the already-compromised gut barrier integrity. Notably, among the above-mentioned alkaloids, nine-tenths of natural compounds exhibit potent protective effects to reduce colonic damage by inhibiting colonic inflammatory status as evidenced by inhibiting the production of pro-inflammatory mediators/cytokines and the populations of innate and adaptive immune cells, such as M1 macrophages, Th1 and Th17 cells. Studies have also shown that the compounds norisoboldine (**14**), isatin (**18**), 3,3-diindolylmethane (**20**), pentoxifylline (**25**), and epiisopiloturine (**29**) exhibit potent protective effects to reduce colonic damage by inhibiting colonic oxidative status via reducing colonic lipid peroxidation products MDA, decreasing ROS production, increasing the levels of GSH and enhancing different enzyme activities of SOD, CAT and GSH-Px. Different studies have frequently revealed that alkaloids such as *N*-methylcytisine (**6**), demethyleneberberine (**10**), sanguinarine (**11**), cavidine (**12**), tetrandrine (**15**), caulerpin (**22**), tetramethylpyrazine (**26**), piperine (**30**), and palmitoylethanolamide (**31**) could ameliorate the signs of colonic inflammation by inhibiting the NF-κB signaling pathway, while aloperine (**5**) exhibited a different action mechanism by inhibiting the PI3K/Akt signaling pathway, and oxymatrine (**4**) could work on both signaling pathways. In addition, both boldine (**13**) and tryptanthrin (**19**) could affect the NF-κB and IL-6/STAT3 signaling pathways simultaneously. It has also been revealed that berberine (**7**) could improve the signs of colonic inflammation by regulating multiple mechanisms including inhibition of the NF-κB, MAPK, PI3K/Akt, STAT1 and STAT3 signaling pathways. In addition, studies have reported that norisoboldine (**14**) could suppress multiple signaling pathways including NF-κB, MAPK and AhR/Nrf/ROS to reduce the signs of colonic inflammation and the oxidative status.

## Conclusion

This paper summarizes the current findings regarding the anti-colitis activity of plant-derived alkaloids and shows how these alkaloids exhibit significant and beneficial effects in alleviating colonic inflammation. These natural alkaloids are not only promising agents for IBD treatment but are also components for developing new wonder drugs. However, the underlying molecular mechanisms or toxicological evaluation of most plant-derived alkaloids still require much scientific research, and their actual efficacies for IBD patients have not been verified well in field research. Thus, further clinical trials to elucidate the efficacy and safety of plant-derived alkaloids and their correlation with in-built cellular and molecular mechanisms are needed to promote plant-derived alkaloids as IBD remedies in the near future.

## Author Contributions

JP, T-TZ, Y-CH, and H-TX did a literature review and prepared the first draft of the manuscript. Y-CH, XL, YL, and L-JW edited the manuscript and proposed and included some vital modifications. Y-CH, H-TX, and L-JW designed the work and wrote the final edition of the manuscript.

## Conflict of Interest Statement

The authors declare that the research was conducted in the absence of any commercial or financial relationships that could be construed as a potential conflict of interest.
